# VAR, ARIMAX and ARIMA models for nowcasting unemployment rate in Ghana using Google trends

**DOI:** 10.1186/s43067-023-00078-1

**Published:** 2023-02-27

**Authors:** Williams Kwasi Adu, Peter Appiahene, Stephen Afrifa

**Affiliations:** 1 Present Address: University of Energy and Natural Resources, Sunyani, Ghana; 2 Tianjin University, Tianjin, China

## Abstract

The analysis of the high volume of data spawned by web search engines on a daily basis allows scholars to scrutinize the relation between the user’s search preferences and impending facts. This study can be used in a variety of economics contexts. The purpose of this study is to determine whether it is possible to anticipate the unemployment rate by examining behavior. The method uses a cross-correlation technique to combine data from Google Trends with the World Bank's unemployment rate. The Autoregressive Integrated Moving Average (ARIMA), Autoregressive Integrated Moving Average with eXogenous variables (ARIMAX) and Vector Autoregression (VAR) models for unemployment rate prediction are fit using the analyzed data. The models were assessed with the various evaluation metrics of mean absolute error (MAE), root mean square error (RMSE), mean absolute percentage error (MAPE), median absolute error (MedAE), and maximum error (ME). The average outcome of the various evaluation metrics proved the significant performance of the models. The ARIMA (MSE = 0.26, RMSE = 0.38, MAE = 0.30, MAPE = 7.07, MedAE = 0.25, ME = 0.77), ARIMAX (MSE = 0.22, RMSE = 0.25, MAE = 0.29, MAPE = 6.94, MedAE = 0.25, ME = 0.75), and VAR (MSE = 0.09, RMSE = 0.09, MAE = 0.20, MAPE = 4.65, MedAE = 0.20, ME = 0.42) achieved significant error margins. The outcome demonstrates that Google Trends estimators improved error reduction across the board when compared to model without them.

## Introduction

The vast amount of information provided by the internet such as Google [1, 2], Twitter [3], social media [4], or combinations of web-based data sources [5, 6] have necessitated its numerously used in recent decades to find the potential of digital information for predictions in a wide range of sectors. Study reviews that Google handles over 92% of all online search requests in the world [7], and has demonstrated to be valid [8], valuable [9], accurate [10], and beneficial [11] for predictions. Google Trends has proven to be a dependable source of trend data for online searches and it is being extensively used by researchers around the world mostly for a real-time prediction of macroeconomic trends [12, 13].

Information that people provide through the internet describes the current state of the people and offers a good understanding mostly of the economic processes, particularly unemployment [14, 15]. Upon all these useful online sources with all the availability of high-frequency data and recent technological advancement, statistical information published on unemployment by nations is released with delays and may still be revised [16, 17]. The way of gathering data for unemployment estimation seems exasperating making it impossible to know how the economy is performing right now but only how it was several months or years ago. This challenge is almost common in all countries, with Ghana not an exemption. This results in Policymakers making assessments in real-time using inadequate information, and knowing the present unemployment state which could help them better understand whether an economy is contracting or expanding and respond [18]. This paper tackles the case by using real-time Google trends data for prediction of unemployment claims in Ghana.

According to the Ghana Statistical Service's most recent census, Ghana's UnEmployment Rate (UER) increased to 13.4% in 2021, up from 6% in 2010, with 32.8% of Ghanaians aged 15 to 24 unemployed. Ghana faces a desperate downturn in economy, and the economy robust growth over the last two decades has not converted into job creation or improved employment circumstances [19]. This unfortunate situation and pressure on jobs have resulted in the loss of hundreds of jobs [20]. It would be of communal interest to produce real-time estimates of the unemployment rate to help policy making to produce real-time unemployment rate. The novelty of this paper is as follows:

- this is the current paper that considers the use of ARIMA, ARIMAX, and VAR in predicting unemployment rate in Ghana.
- the paper considers Google Trends indicators to predict unemployment rate in Ghana, which in turn can be used for the West African sub region.
- the paper is the current to consider unemployment rate predictions in the literature.
- the current paper provides the strategies and benchmarks for governments, agencies and organizations to make informed decisions on unemployment in Ghana, Africa, and the world as a whole.

The rest of the paper is organized as follows: The next section discusses related literature on forecasting using online search data. Section “Methodology” describes the methodology used for identifying a large number of keywords that may help in the prediction of unemployment claims, also provides a brief overview of the models used for comparison of results. The results of the models are discussed in section “Results and discussion.” Section “Conclusion and future works” gives the conclusion and discusses the importance of using different categories of keywords for the prediction of the unemployment claims.

## Related works

Online search engines are frequently used for real-time research. Due to the huge amount of daily search queries, Ettredge et al. [21] took the first initiative by first looking into how real-time forecasting may be done by using the Internet and the study's findings reveal a strong link between Internet-related web search activity and unemployment rate in the USA [22, 23] continued by looking at how web search data, particularly Google, could be utilized to improve forecasting of a range of economic parameters, such as jobless claims, retail sales, real estate demand, and vacation destination preferences. Several studies of real-time forecasting utilizing internet data, particularly Google Trends (GT) data, have been published since these papers, but this work focuses on unemployment prediction.

To anticipate UERs during the COVID-19 pandemic in Indonesia, Rizky et al. [2] used GT data query share for the keyword "phk" (work termination) and earlier series from the official labor force survey performed by Badan Pusat Statistik (Statistics Indonesia). As a result of using the GT index query as an exogenous variable to capture current conditions of a phenomenon that is occurring, results of predicting open UER using ARIMAX during the COVID-19 period generate forecast values that are reliable and near to reality. Petropoulos et al. [24] used text mining algorithms to develop a financial lexicon based on a collection of 10,000 Central Bank speeches. Google inquiries, according to experts, can predict future market volatility in a short time (one month). Tuhkuri [25] used the ETLAnow model and no Google search data to estimate official UER in the European Union (EU) - 28 countries. Google Inc.'s Google Trends database, as well as Eurostat's Labor Force Statistics, are the model's primary data sources. Findings suggest that Google searches are linked to the EU UER, even after controlling for country-level, delayed, and seasonal effects.

Tuhkuri [26] used GT's database from Google Inc. and Labor Force Statistics from the Current Population Survey and US Bureau of Labor Statistics. Results reveal that Google searches' predictive ability is inadequate for short-term forecasting, that the utility of Google data for forecasting purpose is occasional, and forecasting accuracy increases are relatively modest. Mulero and García-Hiernaux [1] used data from GT and the Spanish State Employment Service to examine a large number of potential explanatory factors for UERs. The results reveal an increase in expected accuracy of 10% to 25%.

Lasso and Snijders [27] adopted GT method to forecast Brazil's UER. The findings reveal that Google search volumes for job-related phrases have significant predictive power, with biweekly search data forecasting the direction of the UER with over 80% accuracy, exceeding baseline methodologies based on seasonal trends by over 15%. Brake and Ramos [28] estimate the UER in the Netherlands using a variable based on the amount of Google search keywords. The predictive capability of the Google Indicator is determined by comparing the accuracy of a benchmark model to an upgraded model with the Google Indicator. According to the statistics, the Google improved models produce up to 27.8% more accurate estimations when considering a one-month forecast horizon.

Simionescu and Zimmermann [14] looked into how internet usage information is used in various industries, with unemployment modeling being a particular area of interest. The results of the research show that there is a lot of potentials that should be investigated further. A vast majority of nations base their unemployment estimation and modeling on internet data. However, the forecast's accuracy is based on each country's internet penetration, the age distribution of online users, and the stability of the generated internet variables. Maas [29] studied if Google search data, and other more traditional predictor elements, may be utilized to anticipate the UER in the USA. The findings indicate that GT forecasting methods proposed in this study are most beneficial in short term.

Jung and Hwang [30] constructed unemployment prediction models for specific age groups using Google search queries related to them (the 30s and 40s) and known unemployment statistics from Statistics Korea. The findings demonstrate that employing a web search query to improve unemployment prediction models for Korea is still useful. Smit [31] investigates whether and to what extent Google search data may be utilized to forecast the US UER. They concluded that GTs enhances the anticipated accuracy of all currently used forecasting approaches.

## Methodology

**Fig. 1:**
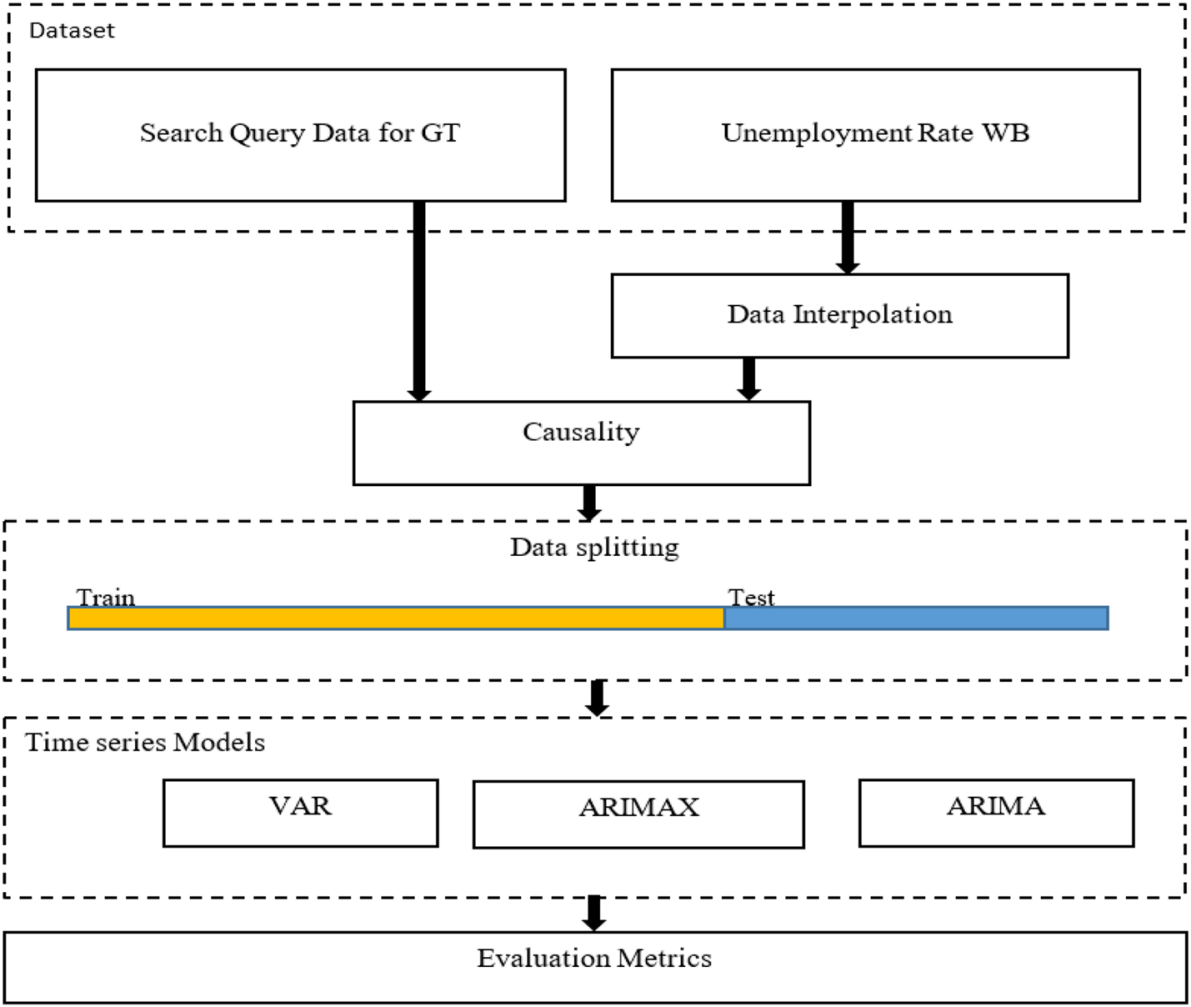
Work flow of experimental design

The study explored the effectiveness of the Google trends by adopting several testing techniques. Figure 1 displays a detailed procedure for the experiment. The steps below are a detailed explanation of Fig. 1.

1. To start, data from GT were joint with interpolated World Bank (WB) UER data to create a single, special dataset for the visualization and study of UER in Ghana using Granger causality.
2. Time Series (TS) data are split into training and test sets after input.
3. Training sets and test sets were used to train and evaluate the models (ARIMA, ARIMAX, and VAR).

### Data

The World Bank (WB) and Google Trends (GT) provided the data for this collection. Google launched the website Google Trends for search analysis in 2006. GT offers a search trend that starts with the year 2004 and shows the frequency with which a certain search phrase is entered into Google's search engine over time about the site's overall search traffic.

GT shows changes in internet interest for any TS in any nation or location over a selected period of time, such as one year, several years, four months, three weeks, thirty days, seven days, four hours, or one hour. Additionally, several sentences from various places can be compared simultaneously. The GT and World Bank data can be downloaded in ".csv" format. In short, GT calculates the number of searches represented mathematically in equation 1, 2, and 3 as follows [32]:

1 $$S(e{)}_{tot,m}={\sum }_{k=1}^{\infty }s(e{)}_{k,m}$$

2 $$Qs(e{)}_{i,m}=\frac{S(e{)}_{i,m}}{S(e{)}_{tot,m}}$$

3 $$\mathrm{RSV}(e{)}_{i,m}=100*\frac{Qs(e{)}_{i,m}}{Qs(e{)}_{\mathrm{max},m}}$$

where *i* = Terms or expressions of the study, *k* = possible terms to search on Google, *m* = months of the study. Additionally, $S(e{)}_{\mathrm{tot},m}$ = total search on Google for one-month m in a particular country, $S(e{)}_{i,m}$ = total searches on Google for a term i of our study for a month m and a country, $Qs(e{)}_{t,m}$ = Query share of a term in a certain month and country, and $\mathrm{RSV}(e{)}_{t,m}$ = Relative search volume of a term in a certain month and country.

Our sample of search terms comprises 50 Google Trends which have been chosen based on the methodology as shown in Table 1. Our data window is restricted to begin with 2010–2020 - since this is the earliest data point for which Ghanaian migrated to using internet. The variable of interest is the unemployment rate date for Ghana downloaded from the World Bank website.

**Table 1:** GT Keywords (50)

Acceptance letter	Distance education	Graduation	Loan application	School admission
American lottery	Distance learning	Health insurance	Mining jobs	Trade
Application for employment	Employment letter	How to make money	Nursing schools	Trading
Application letter	Employment	How to start a business	Nursing training	Training college
Business opportunities	Entrepreneur	Income tax	Online application	Unemployment
Career	Exchange rate	Job application	Online jobs	USA jobs
Companies in Ghana	Foreign exchange	Job interview	Online money	USA visa
Cover letter	Ghana economy	Job opportunities	Online schools	Vacancy
Curriculum vitae	Ghana jobs	Jobs in Ghana	Police recruitment	Visa application
Cv	Graduate	Jobs in USA	Scholarship	Visa

### Interpolation

For extracting high-frequency data (such as monthly or weekly data) from low-frequency data (such as annual data), the Chow-Lin approach, a disaggregation method, is utilized [33]. The method makes sure that the high-frequency series' average, first, and last values correspond to those of the low-frequency series. The following two-step additive structure is the general temporal disaggregation framework for developing a high-frequency estimate, according to [33]. Equation 4 describes the Chow-Lin approach.

4 $${\upsilon }_{j}={\overline{\upsilon }}_{j}+{\sum }_{i=1}^{n}{F}_{ji}\left({y}_{i}-{\sum }_{q=1}^{m}{H}_{iq}{\overline{\upsilon }}_{q}\right)$$

Make a preliminary high-frequency series ${\overline{\upsilon }}_{j}$ using auxiliary data from several indicator series. To incorporate this data, a generalized least squares regression strategy is frequently utilized. Analyze the differences in residuals between the observed low-frequency series and the high-frequency series that have been aggregated to the low-frequency scale (through the matrix $H\in {f}^{n\times m}$). Then, create a temporally consistent high-frequency version ${y}_{i}$ by distributing these differences among the high-frequency periods using the distribution matrix $F\in {R}^{n\times m}$.

### Causality (granger causality (GC))

GC test examines the connection between the current value of one variable and the historical values of another variable to find a causal direction between two or more time series [34]. According to [35] GC indexes of two series *Y* and *X* can be computed by finding the variance of the error samples. If *X* and *Y* are independent, then *X*($var(\varepsilon )$) = *Y*($var(\varepsilon )$), where $var(\varepsilon )$ denotes the variance of the error *e*. Otherwise, the two equations do not hold. For example, if *X* is the cause of *Y*, then *X* ($v\mathit{ar}(\varepsilon )$) > *Y* ($v\mathit{ar}(\varepsilon )$). It can be represented by the formula in Eq. 5 [36]

5 $$\begin{aligned} F_{{\left( {X \to Y} \right)}} & = \log \frac{{X_{{\left( {var(\varepsilon } \right))}} }}{{Y_{{\left( {{\text{var}} (\varepsilon } \right))}} }} \\ F_{{\left( {Y \to X} \right)}} & = \log \frac{{Y_{{\left( {var(\varepsilon } \right))}} }}{{X_{{\left( {{\text{var}} (\varepsilon } \right))}} }} \\ \end{aligned}$$

If ${F}_{(X\to Y)}$ ≥ 0 and ${F}_{(Y\to X)}$ ≥ 0 then the indexes of causality can be analyzed. Specifically, if ${F}_{(X\to Y)}$ >${F}_{(Y\to X)}$, then X is the cause of Y, or the information flowing from *X* to *Y* is more than that from Y to X; if ${F}_{(X\to Y)}$ <${F}_{(Y\to X)}$, then Y is the cause of *X*.

### Training, and test

The overall data set was split into training and test data sets with the shares close to 80% from 2010 to 2018 dataset, with the remaining 20% from 2019 to 2020 designated for testing. Table 2 shows specific splitting procedure that divides the dataset. In the second step, the test set of two years frames is further divided into yearly (Y1), half-year, quartile, and monthly such that UER was tested in the different time frames.

**Table 2:** Training, Test Sample

Data / train / Test	Year(s)	GT (50)	UER %	Percentage %
Total size 2010–2020	10 years	574	574	100% of data size
Training 2010–2018	8 years	470	470	80% of data size
Test set 2019–2020	2 years (*Y*_24/12_)	104	104	20% of data size (100% of test set)
	1 year 9 months (*Y*_21/12_)	91	91	88% of test set
	1 year 6 months (*Y*_18/12_)	78	78	75% of test set
	1 year 3 months (*Y*_15/12_)	65	65	63% of test set
	1 year (*Y*_12/12_)	52	52	50% of test set
	9 months (*Y*_9/12_)	39	39	38% of test set
	6 months (*Y*_6/12_)	26	26	25% of test set
	3 months (*Y*_3/12_)	13	13	13% of test set
	1 month (*Y*_1/12_)	4	4	4% of test set

### Models

The data science project of TS forecasting is crucial for many processes that happen over time. TS forecasting is a practical method for figuring out how past data influence present results. Making short- and long-term projections and pattern-spotting using previous data allows for this. The TS used were ARIMA, VAR and ARIMAX.

#### VAR

VAR is a forecasting method that can be used when two or more TS interact. In other words, the TS in question has a two-way relationship. VAR models can be used to assess and predict multivariate TS data, which sets them apart from univariate autoregressive models. VAR models are often used in economics. For a VAR model with a large number of interconnected TS variables. Equation 6 represents the VAR model

6 $$\left[\begin{array}{c}{y}_{1}\\ {y}_{2}\\ \vdots \\ {y}_{n}\end{array}\right]=\left[\begin{array}{c}{c}_{1}\\ {c}_{2}\\ \vdots \\ {c}_{n}\end{array}\right]+\left[\begin{array}{c}{\phi }_{11}\cdots \\ {\phi }_{21}\cdots \\ \vdots \\ {\phi }_{n1}\cdots \end{array}\right]\left[\begin{array}{c}{y}_{1,t-1}\\ {y}_{2,t-1}\\ \vdots \\ {y}_{n,t-1}\end{array}\right]+....+\left[\begin{array}{c}{\phi }_{1,t-p}...\\ {\phi }_{2, t-p}\cdots \\ \vdots \\ {\phi }_{n, t-p}\cdots \end{array}\right]\left[\begin{array}{c}{y}_{1,t-p}\\ {y}_{2,t-p}\\ \vdots \\ {y}_{n,t-p}\end{array}\right]+\left[\begin{array}{c}{\varepsilon }_{1}\\ {\varepsilon }_{2}\\ \vdots \\ {\varepsilon }_{n}\end{array}\right]$$

where the c is the intercept, $\phi$ coefficient of lags of y till order p, and ɛ error. Here, it is shown as a system of equations with one equation per TS variable. VAR is adaptable, requires less time and information [37], and makes it simple to integrate additional data [38]. VAR models, however, have the drawback of being unable to take into account when the measure of the dispersion between numbers in a data set changes across various time series values [39].

#### ARIMA

ARIMA combines the ideas of autoregression and moving average to provide forecasts that are linear combinations of previous variable values and forecast errors. ARIMA is characterized by three factors: *p*, *d*, and *q* signify the number of lagged (or previous) data to consider for autoregression, the number of times the raw observations are differenced, as well as size of the moving average window, respectively.

The forecasting equation is structured in Eq. 7 as follows:

7 $${F}_{t}={L}_{t}+{\Omega }_{1}{D}_{t-1}^{^{\prime}}+\dots +{\Omega }_{p}{D}_{t-p}^{^{\prime}}+{\beta }_{q}{E}_{t-1}+\cdots +{\beta }_{q}{E}_{t-q}$$

where ${F}_{t}$ = forecast point at time *t*, ${L}_{t}$ = Level at time t (straight line approximation of all your data at one time point—calculated in ARIMA, it uses the mean of differenced data time smoothing constants), ${D}_{t-p}^{`}$ = Previous difference observed data points, ${E}_{t-q}$= Error in prediction on previous data points, and $\Omega$ and *β* are smoothing constants.

Many scholars who used time series recently explored ARIMA. However, the ARIMA model only applies to one variable, does not adequately describe some data turning points, and cannot adequately convey relationships between variables [40, 41]. As a result, it is insufficient to describe genuine issues.

#### ARIMAX

The ARIMAX model is an extension of the ARIMA model. The model includes other independent variables that are the X added to the end and stands for “exogenous variables.” This involves adding a separate different outside variable to help measure our endogenous variable.

Equation 8 is structured as follows:

8 $$\Delta Pt = c + \beta X + \phi 1\Delta Pt - 1 + \theta 1 \, \smallint t - 1 + \smallint t$$

where *Pt* and *Pt*−1 represent the values in the current period and 1 period ago, respectively. Similarly, ϵ*t* and ϵ*t*−1 are the error terms for the same two periods. *C* is just a baseline constant. *ϕ*1 and *θ*1, express what parts of the value *Pt*−1 and error ϵ*t*−1 last period are relevant in estimating the current one. *β* is a coefficient which will be estimated based on the model selection and the data. *X* is the exogenous variable of interest. ARIMAX is helpful since it combines the time series and regression components into one model. However, it can be challenging to interpret the independent variable that may have an impact on the result.

### Evaluation metrics

We compute the mean squared error (MSE), mean absolute error (MAE), root mean squared error (RMSE), mean absolute percentage error (MAPE), median absolute error (MedAE), and maximum error (ME) to assess the forecasting accuracy of each model. Equations 9, 11, 12, 13, 14, and 14 represent the aforementioned evaluation metrics.

9 $$\mathrm{MSE}=\frac{1}{n}{\sum }_{i=1}^{n}({y}_{i}-{\widehat{y}}_{i}{)}^{2}$$

10 $$\mathrm{MAE}=\frac{{\sum }_{i=1}^{n}\left|{y}_{i}-{\widehat{y}}_{i}\right|}{n}$$

11 $$\mathrm{RMSE}=\sqrt{\frac{1}{n}{\sum }_{i=1}^{n}({y}_{i}-{\widehat{y}}_{i}{)}^{2}}$$

12 $$\mathrm{MAPE}=\frac{100}{n}{\sum }_{i=1}^{n}\left|\frac{{y}_{i}-{\widehat{y}}_{i}}{{y}_{i}}\right|$$

13 $$\mathrm{MedAE}=\mathrm{median}\left(\left|y-\widehat{{y}_{i}}\right|,...,\left|{y}_{n}-{\widehat{y}}_{n}\right|\right)$$

14 $$\mathrm{ME}=\mathrm{max}\left(\left|y-\widehat{{y}_{i}}\right|,...,\left|{y}_{n}-{\widehat{y}}_{n}\right|\right)$$

where *y* denotes current UER and $\widehat{y}$ is expected UER. Our study used six (6) different valuation metrics to evaluate the models. By employing more evaluation metrics, we were able to choose the optimum strategy while also confirming that each model was able to complete the underlying predicting task.

## Results and discussion

### Interpolation

**Fig. 2:**
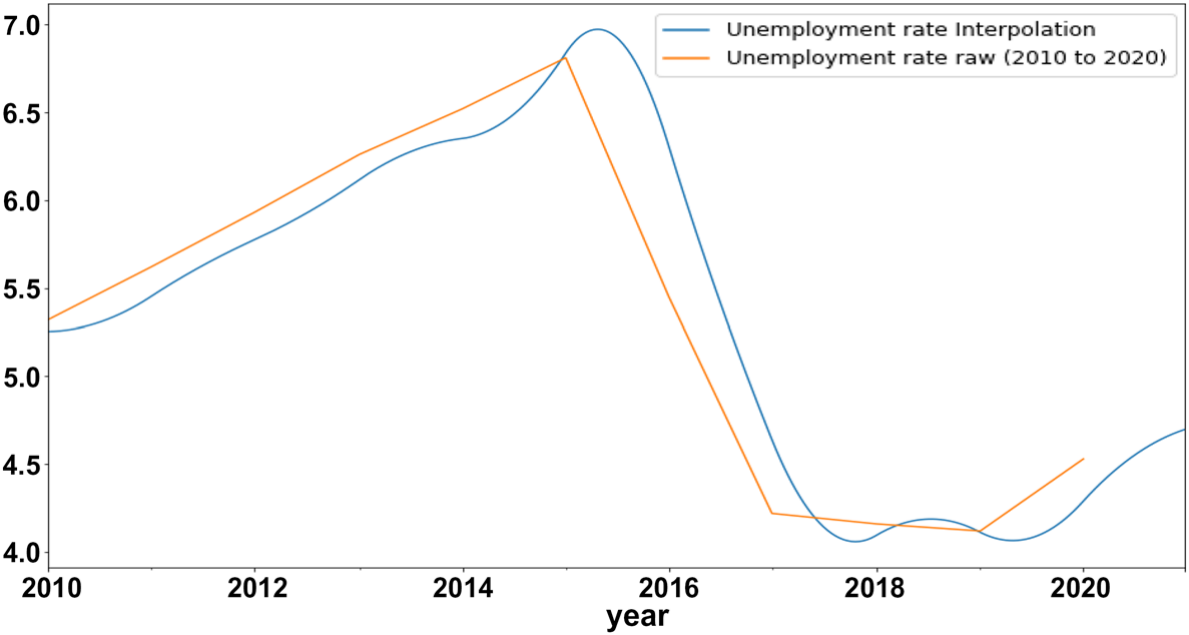
Unemployment rate and an interpolation unemployment rate

The basic goal of temporal disaggregation methods is to create a new TS while preserving the short-term behavior of higher frequency indicator series. This TS must be coherent with low-frequency data. For the UER and interpolated UER in question, standard descriptive data are provided in Table 3. The table demonstrates that the UER and the interpolated UER are nearly equal. Visual representations of the UER and interpolated UER are shown in Fig. 2.

**Table 3:** Descriptive statistics for UER and Interpolated UER

Statistics	Unemployment rate	interpolation unemployment rate
Count	11.000000	574.000000
Mean	5.358182	5.357195
Std	0.981140	0.953488
Min	4.120000	4.059191
25%	4.375000	4.305773
50%	5.450000	5.446064
75%	6.095000	6.218123
max	6.810000	6.971820

The graph shows that the interpolated unemployment rate, which comes from a dataset of 574 recordings, and the actual unemployment rate, which comes from a dataset of 11 records, both vary in the same way over time, proving that our dataset is equal in mean and standard deviation.

### Cross-correlation function (CCF) analysis

Table 4 outlines the keywords whose trends were highly linked with UER using Granger causality Test (GCT). We compiled a list of terms from Table 4 with a high p for lag << 1 0.05 that are associated with the UER.

**Fig. 3:**
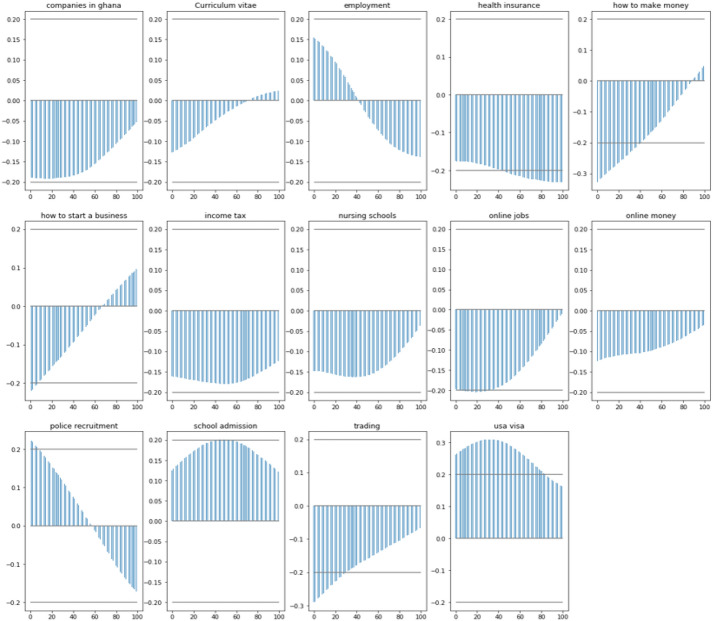
GCT analysis results of selected GT (*x*) and WB UER (*y*) keywords

The table demonstrates how 14 of the 50 GT (x1 to 50) estimators for Ghana are related to the WB UERs series (*y*). The cells in the table with *p*(*v*) values that are less than 0.05 for the first lag were chosen. Figure 3 displays a graph of GT estimators with p0.05 analysis results. The graph shows that there is a range of correlations between +1 and -1, where +1 represents the total positive correlation, 0 represents the absence of any correlation, and -1 represents the total negative correlation. The lags and past values of the 14 indicators are statistically significant in the equation and predicting the future values of unemployment rate.

**Table 4:** GCT Analysis

Granger causality Test (squared residual (SSR) based *F* test)				
Acceptance letter Lag 1 * p*(*v*): 0.2038 Lag 2 * p*(*v*): 0.0041	Distance education Lag 1 * p*(*v*): 0.2601 Lag 2 * p*(*v*): 0.1064	Graduation Lag 1 * p*(*v*): 0.0968 Lag 2 * p*(*v*): 0.4066	Loan application Lag 1 * p*(*v*): 0.2634 Lag 2 * p*(*v*): 0.0340	School admission Lag 1 * p*(*v*): **0.0393** Lag 2 * p*(*v*): 0.0214
American lottery Lag 1 * p*(*v*): 0.7104 Lag 2 * p*(*v*): 0.8864	Distance learning Lag 1 * p*(*v*): 0.5602 Lag 2 * p*(*v*): 0.8839	Health insurance Lag 1 * p*(*v*): **0.0019** Lag 2 * p*(*v*): 0.0574	Mining jobs Lag 1 * p*(*v*): 0.7852 Lag 2 * p*(*v*): 0.1818	Trade Lag 1 * p*(*v*): 0.3928 Lag 2 * p*(*v*): 0.7702
Application for employment Lag 1 * p*(*v*): 0.1214 Lag 2 * p*(*v*): 0.0350	Employment letter Lag 1 * p*(*v*): 0.4170 Lag 2 * p*(*v*): 0.5553	How to make money Lag 1 * p*(*v*): **0.0000** Lag 2 * p*(*v*): 0.0000	Nursing schools Lag 1 * p*(*v*): **0.0125** Lag 2 * p*(*v*): 0.1505	Trading Lag 1 * p*(*v*): **0.000** Lag 2 * p*(*v*): 0.000
application letter Lag 1 * p*(*v*): 0.9501 Lag 2 * p*(*v*): 0.1191	Employment Lag 1 * p*(*v*): **0.0279** Lag 2 * p*(*v*): 0.0353	How to start a business Lag 1 * p*(*v*): **0.0000** Lag 2 * p*(*v*): 0.0000	Nursing training Lag 1 * p*(*v*): 0.2080 Lag 2 * p*(*v*): 0.4708	training college Lag 1 * p*(*v*): 0.4841 Lag 2 * p*(*v*): 0.4856
Business opportunities Lag 1 * p*(*v*): 0.5516 Lag 2 * p*(*v*): 0.4648	Entrepreneur Lag 1 * p*(*v*): 0.4588 Lag 2 * p*(*v*): 0.1039	Income tax Lag 1 * p*(*v*): **0.0012** Lag 2 * p*(*v*): 0.0156	Online application Lag 1 * p*(*v*): 0.4383 Lag 2 * p*(*v*): 0.3724	Unemployment Lag 1 * p*(*v*): 0.0970 Lag 2 * p*(*v*): 0.1388
Career Lag 1 * p*(*v*): 0.0843 Lag 2 * p*(*v*): 0.0015	Exchange rate Lag 1 * p*(*v*): 0.7634 Lag 2 * p*(*v*): 0.7559	Job application Lag 1 * p*(*v*): 0.1591 Lag 2 * p*(*v*): 0.0143	Online jobs Lag 1 * p*(*v*): **0.0001** Lag 2 * p*(*v*): 0.0036	USA jobs Lag 1 * p*(*v*): 0.2268 Lag 2 * p*(*v*): 0.2227
companies in Ghana Lag 1 * p*(*v*): **0.0031** Lag 2 * p*(*v*): 0.0534	Foreign Exchange Lag 1 * p*(*v*): 0.1086 Lag 2 * p*(*v*): 0.0025	Job interview Lag 1 * p*(*v*): 0.0815 Lag 2 * p*(*v*): 0.0443	Online money Lag 1 * p*(*v*): **0.0322** Lag 2 * p*(*v*): 0.1039	USA visa Lag 1 * p*(*v*): **0.0000** Lag 2 * p*(*v*): 0.0000
Cover letter Lag 1 * p*(*v*): 0.1414 Lag 2 * p*(*v*): 0.1310	Ghana economy Lag 1 * p*(*v*): 0.7133 Lag 2 * p*(*v*): 0.8431	Job opportunities Lag 1 * p*(*v*): 0.2461 Lag 2 * p*(*v*): 0.0003	Online schools Lag 1 * p*(*v*): 0.3268 Lag 2 * p*(*v*): 0.5662	Vacancy Lag 1 * p*(*v*): 0.7254 Lag 2 * p*(*v*): 0.0453
Curriculum vitae Lag 1 * p*(*v*): **0.0135** Lag 2 * p*(*v*): 0.0130	Ghana jobs Lag 1 * p*(*v*): 0.1970 Lag 2 * p*(*v*): 0.0301	Jobs in Ghana Lag 1 * p*(*v*): 0.1561 Lag 2 * p*(*v*): 0.0300	police recruitment Lag 1 * p*(*v*): **0.0026** Lag 2 * p*(*v*): 0.0004	Visa application Lag 1 * p*(*v*): 0.3595 Lag 2 * p*(*v*): 0.1187
Cv Lag 1 * p*(*v*): 0.8731 Lag 2 * p*(*v*): 0.0609	Graduate Lag 1 * p*(*v*): 0.1661 Lag 2 * p*(*v*): 0.3506	Jobs in USA Lag 1 * p*(*v*): 0.4430 Lag 2 * p*(*v*): 0.0679	Scholarship Lag 1 * p*(*v*): 0.4433 Lag 2 * p*(*v*): 0.0009	Visa Lag 1 * p*(*v*): 0.1565 Lag 2 * p*(*v*): 0.1066

The bold values are the estimators whose * p*-value were lesser than 0.05. This was used as a benchmark for the study

### Model result

According to the experimental design aforementioned, detailed experiments with different TS models were conducted using univariate or multivariate models. The models and order utilized in building series are ARIMA (1, 2, 1), ARIMAX (4, 1, 3) and VAX (3, 0). Table 5 illustrates the data evaluation metrics for the Models.

**Table 5:** Evaluation results over the TS models for the selected periods

	Model name	MSE	RMSE	MAE	MAPE	MedAE	ME
*Y*_1/12_	ARIMA	0.00	0.00	0.00	0.19	0.00	0.00
	ARIMAX	0.00	0.00	0.00	0.18	0.00	0.00
	VAR	0.00	0.00	0.00	0.04	0.00	0.00
*Y*_3/12_	ARIMA	0.00	0.02	0.02	0.69	0.01	0.04
	ARIMAX	0.00	0.00	0.01	0.67	0.01	0.04
	VAR	0.00	0.00	0.02	0.40	0.01	0.04
*Y*_6/12_	ARIMA	0.01	0.08	0.06	1.76	0.05	0.18
	ARIMAX	0.01	0.01	0.06	1.70	0.04	0.17
	VAR	0.01	0.01	0.06	1.45	0.05	0.16
*Y*_9/12_	ARIMA	0.03	0.18	0.14	3.44	0.10	0.39
	ARIMAX	0.03	0.03	0.13	3.36	0.10	0.38
	VAR	0.02	0.02	0.12	2.91	0.10	0.31
*Y*_12/12_	ARIMA	0.10	0.31	0.24	5.78	0.18	0.67
	ARIMAX	0.09	0.09	0.23	5.67	0.18	0.66
	VAR	0.06	0.06	0.19	4.58	0.16	0.49
*Y*_15/12_	ARIMA	0.22	0.47	0.36	8.57	0.28	0.99
	ARIMAX	0.21	0.21	0.35	8.40	0.28	0.97
	VAR	0.11	0.11	0.27	6.29	0.24	0.63
*Y*_18/12_	ARIMA	0.41	0.64	0.49	11.51	0.40	1.28
	ARIMAX	0.39	0.39	0.48	11.23	0.39	1.23
	VAR	0.17	0.17	0.34	7.75	0.32	0.70
*Y*_21/12_	ARIMA	0.64	0.80	0.62	14.43	0.53	1.55
	ARIMAX	0.60	0.60	0.60	13.98	0.53	1.47
	VAR	0.22	0.22	0.39	8.85	0.40	0.71
*Y*_24/12_	ARIMA	0.92	0.96	0.76	17.27	0.69	1.79
	ARIMAX	0.92	0.92	0.76	17.27	0.69	1.79
	VAR	0.25	0.25	0.43	9.57	0.49	0.71
Average	ARIMA	0.26	0.38	0.30	7.07	0.25	0.77
	ARIMAX	0.25	0.25	0.29	6.94	0.25	0.75
	VAR	0.09	0.09	0.20	4.65	0.20	0.42

#### Evaluation of the models

The selected significant prospective determinants of the unemployment rate are taken into account with the aid of various evaluation metrics. Consideration was given to the significant y chosen for the unemployment rate in all periods. Table 5 provides an overview of the performance metrics MSE, RMSE, MAE, MAPE, MedAE, and ME for all the periods. The results show that over the first five measurement periods, the model was able to forecast with little error. Additionally, for all models, the error margin rises as the anticipated period grows. Furthermore, for nearly all periods and virtually all evaluation techniques, VAR was able to forecast with minimum error.

**Fig. 4:**
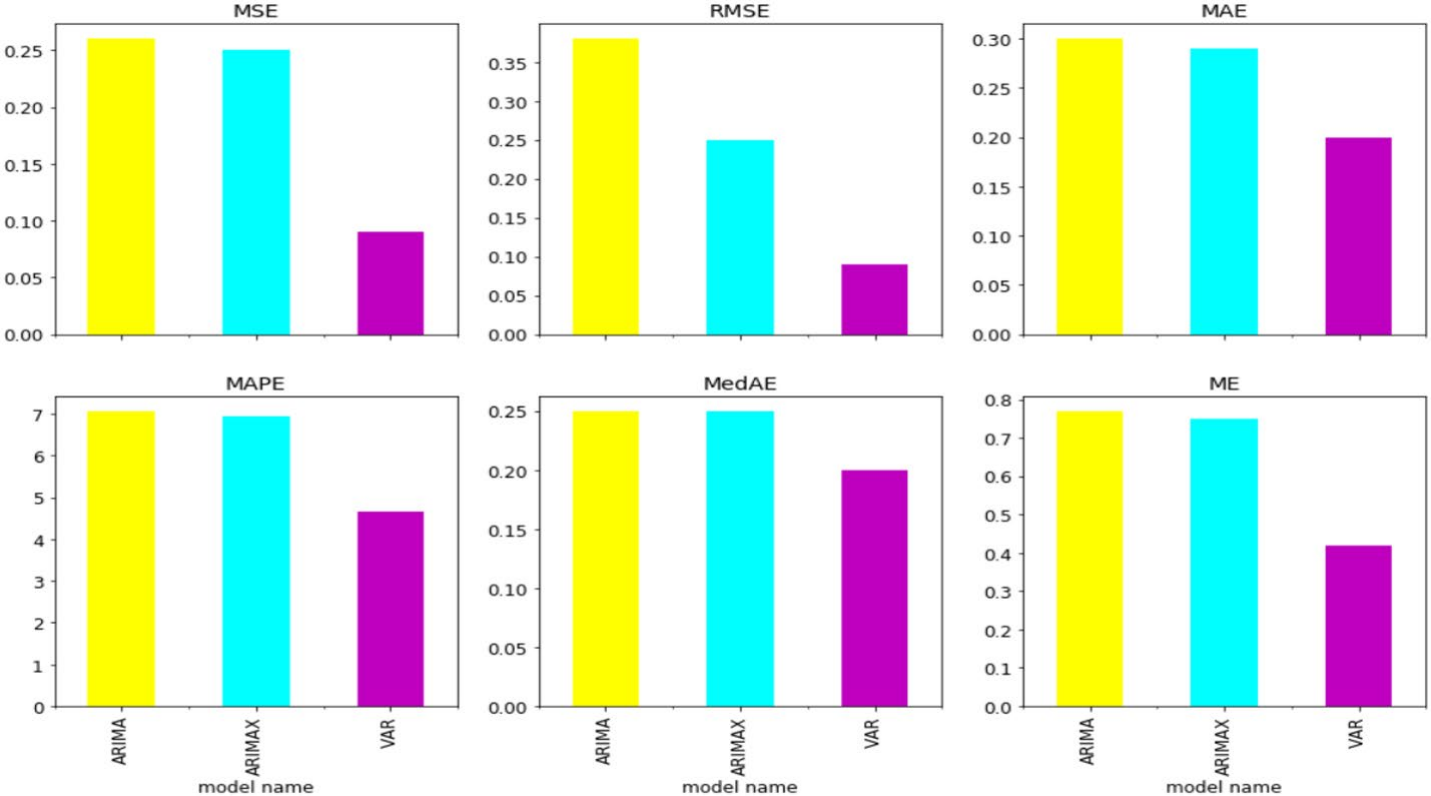
Visualization comparison of the average evaluation result for the models

We created an average based on each evaluation metric results for all models, as shown in Table 5, to decide and choose the best models for the forecast. The VAR model had the best and least average error values, with MSE = 0.09, RMSE = 0.09, MAE = 0.20, MAPE = 4.65, MedAE = 0.20, and ME = 0.42, as demonstrated by the average findings in Fig. 4. This demonstrated how better the proposed model VAR (multivariate TS) with GT estimators is compared to ARIMA and ARIMX. The VAR was able to detect a minor growth even if the models did not follow the major trend of UER change. The graph demonstrated how much better and more effective the VAR model is than the other models.

**Fig. 5:**
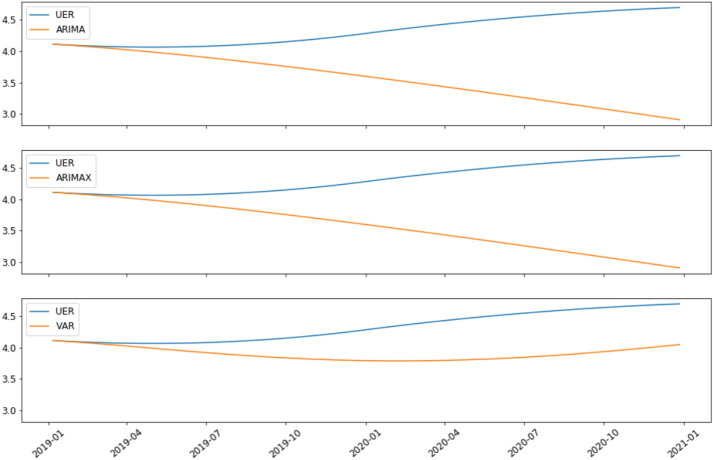
Real UER and forecasted UER for Ghana for the 2 years over Models

Figure 5 shows the actual UER for Ghana as well as the predicted visualization for each of the models over the two-year timeframe. Except for VAR, which is somewhat in line and reflected the modest shift, all models were not in line with the UER, according to the figure. The VAR Model outperforms all other models (ARIMA and ARIMAX). Most models in economic condition approximation perform well in a stable environment, but they lack the prudence to foresee hidden economic change. In both steady and dynamic settings, the VAR Model linking input factors derived from rich high-frequency timely variables for predicting UER perform better.

## Conclusion and future works

The issue is not a dearth of data, but rather a dearth of information that can be used for planning, strategy, and decision-making. Using big data, such as Google Trends, can assist the entire government system. Google Trends provides access to a huge unfiltered collection of actual Google search requests. People use Google for a wide range of informational and topical searches, making it a valuable search engine. 50 words or phrases were of interest. Google Trends (GT) search query data were used to derive values for search relating to Jobs, society, social services, and economic indicators. The study identified a number of factors that influence the unemployment rate, including "how to make money," "how to start a business," "jobs in Ghana," "jobs in the USA," "online money," "nurse application," "visa application," and "police recruiting." This study proposes a technique to first implementing pre-processing to overcome the difficulty of handling the vast data and describes an in-depth look into the use of ARIMA, ARIMAX and VAR in nowcasting unemployment in Ghana as a use-case.

In terms of prediction accuracy, error margin, and model reliability, results show that the VAR method surpassed all other techniques. VAR (MSE = 0.09, RMSE = 0.09, MAE = 0.20, MAPE = 4.65, MedAE = 0.20, ME = 0.42) achieved significant error margins. This is compelling evidence that real-time UER forecasting at a daily level of generality is possible. Most models in economic condition approximation perform well in a stable environment, but they lack the prudence to foresee hidden economic change. In both steady and dynamic settings, the VAR Model linking input factors derived from rich high-frequency timely variables for predicting UER perform better. The objective of successful citizen care management can be attained with the use of Google Trends by offering effective data-driven services to citizens and predicting their needs based on the analysis of surveys taken among various groups of citizens. In future, more data will be collected to train with artificial intelligence techniques to generate decision support systems.

In the current study, we have highlighted a few predictor variables that contribute to the nation's unemployment rate and are crucial in figuring out unemployment. The government can also use this study's crucial information to make data-driven decisions. The government will be assisted in strengthening technical and vocational institutions. These will then bring in revenue and be put toward development. Additionally, it will be useful in establishing the state of the economy while formulating monetary policy. We recommend using machine learning model for future work.

## Data Availability

The data presented in this study are publicly available through the Fig Share repository via Afrifa, Stephen (2022): unemployment_data.csv. figshare. Dataset. https://doi.org/10.6084/m9.figshare.20311167.v1.
